# Functional Implications of RFRP-3 in the Central Control of Daily and Seasonal Rhythms in Reproduction

**DOI:** 10.3389/fendo.2019.00183

**Published:** 2019-04-10

**Authors:** Eleni Angelopoulou, Clarisse Quignon, Lance J. Kriegsfeld, Valérie Simonneaux

**Affiliations:** ^1^Institut des Neurosciences Cellulaires et Intégratives (CNRS UPR 3212), Université de Strasbourg, Strasbourg, France; ^2^Netherlands Institute for Neuroscience (NIN), Amsterdam, Netherlands; ^3^Department of Psychology, Helen Wills Neuroscience Institute, University of California, Berkeley, Berkeley, CA, United States

**Keywords:** daily rhythm, seasonal rhythm, clock, melatonin, vasopressin, vasoactive intestinal peptide, kisspeptin, LH

## Abstract

Adaptation of reproductive activity to environmental changes is essential for breeding success and offspring survival. In mammals, the reproductive system displays regular cycles of activation and inactivation which are synchronized with seasonal and/or daily rhythms in environmental factors, notably light intensity and duration. Thus, most species adapt their breeding activity along the year to ensure that birth and weaning of the offspring occur at a time when resources are optimal. Additionally, female reproductive activity is highest at the beginning of the active phase during the period of full oocyte maturation, in order to improve breeding success. In reproductive physiology, it is therefore fundamental to delineate how geophysical signals are integrated in the hypothalamo-pituitary-gonadal axis, notably by the neurons expressing gonadotropin releasing hormone (GnRH). Several neurochemicals have been reported to regulate GnRH neuronal activity, but recently two hypothalamic neuropeptides belonging to the superfamily of (Arg)(Phe)-amide peptides, RFRP-3 and kisspeptin, have emerged as critical for the integration of environmental cues within the reproductive axis. The goal of this review is to survey the current understanding of the role played by RFRP-3 in the temporal regulation of reproduction, and consider how its effect might combine with that of kisspeptin to improve the synchronization of reproduction to environmental challenges.

## RFRP-3 Neurons as Regulators of the Hypothalamo-Pituitary Gonadal Axis

### Functional Organization of the Hypothalamo-Pituitary-Gonadal (HPG) Axis

Mammalian reproduction is tightly controlled by a small set of neurons producing the neuropeptide gonadotropin-releasing hormone (GnRH). These cell bodies are concentrated in specific hypothalamic areas [the preoptic area (POA), the vascular organ of the lamina terminalis and, in non-rodent species, the mediobasal hypothalamus] and project principally to the median eminence where they release GnRH into the anterior pituitary portal blood supply in a pulsatile manner (1). In turn, GnRH stimulates the secretion of the gonadotropins, follicle-stimulating (FSH) and luteinizing (LH) hormones. FSH and LH enter the general circulation to regulate gameto- and steroidogenesis, respectively, in the gonads.

Mechanisms regulating GnRH neurons are thought to involve upstream neuronal inputs. Glutamate and γ-aminobutyric acid fibers, located close to GnRH perikarya and axons, have been shown to stimulate and/or inhibit GnRH release (2–4) Neuropeptide Y-containing fibers also contact a majority of GnRH neurons where they predominantly exert an inhibitory effect on GnRH release (5, 6). Recent studies, however, have highlighted an important role of two hypothalamic neuropeptides, kisspeptin and (Arg)(Phe) related peptide-3 (RFRP-3), in the regulation of GnRH neurons. Kisspeptin expressing neurons are located in two hypothalamic areas: the preoptic area, where they project to GnRH cell bodies to drive the GnRH surge in female mammals, and the arcuate nucleus, where they project principally to GnRH fiber terminals at the median eminence to drive pulsatile GnRH (7). RFRP-3 expressing neurons, mostly located in the dorsomedial hypothalamus (DMH), project to various neuronal populations including GnRH and kisspeptin neurons, yet the effects of RFRP-3 on reproduction seem to vary according to species, sex, and environmental conditions [(8–10) for reviews].

To maintain the reproductive axis within proper functioning limits, sex steroids produced by the gonads feed back to the pituitary and hypothalamus. In males, testosterone acts to suppress GnRH and the gonadotropins through negative feedback whereas, in females, the feedback is more complex with estradiol (E2) having positive or negative feedback effects depending on the stage of the ovarian cycle and its circulating concentration. Specifically, during the follicular phase of the ovulatory cycle, low concentrations of E2 exert negative feedback, whereas upon oocyte maturation, higher concentrations of E2 exert positive feedback, triggering a large release of GnRH in the anterior pituitary portal blood supply which, in turn, induces a surge of LH that initiates ovulation (11). Contrary to early expectation, GnRH neurons do not appear to be the directly responsive to E2 feedback as these cells do not express E2 receptors (ER)α and only express low levels of ERβ (10, 11). Likewise, mice with GnRH neuron-specific deletion of ERβ do not exhibit any gross reproductive dysfunction (12). Therefore, the central structures integrating sex steroid feedback are upstream of GnRH neurons and evidence now indicates that kisspeptin neurons (13, 14) and, to a less and unclear extent, RFRP-3 neurons (13, 14) are relaying gonadal hormone feedback to the reproductive system.

Because reproduction is particularly energetically demanding, it is critical that a number of intrinsic and extrinsic factors contribute to optimizing breeding success and offspring survival. Therefore, the reproductive axis is sensitive to various signals such as metabolic activity, stress level, development stage, hormonal milieu, and geophysical cues. Thus, in female mammals, timing of the preovulatory LH surge is driven by daily signals in addition to positive E2 feedback. Additionally, in seasonal breeders, annual changes in daily light duration (photoperiod) synchronize reproduction with the seasons. Recent studies have highlighted the pivotal role of RFRP-3 neurons, as well as kisspeptin neurons, in relaying both daily and seasonal cues to the HPG axis, particularly to GnRH neurons. The following review will discuss how RFRP-3 regulates mammalian reproduction and contributes to its synchronization with the time of the day and the year.

### The RFRP-3 System

The ortholog of RFRP-3 was originally discovered in birds, with Tsutsui et al. identifying a novel (Arg)(Phe) hypothalamic peptide that inhibited pituitary gonadotropin secretion from cultured quail pituitary (15). Because this peptide selectively inhibited the gonadotropins, without altering other pituitary hormones, the authors named it gonadotropin-inhibitory hormone (GnIH). Subsequent findings indicated that GnIH receptor is expressed in quail pituitary (16, 17) and *in vivo* GnIH administration decreases common α, LHβ, and FSHβ subunit expression (16, 18). In birds, the GnIH precursor cDNA encodes one GnIH and two GnIH-related peptides (GnIH-RP1 and GnIH-RP2) (15, 19). In mammals, the homologous gene encodes three peptides [RFamide-related peptides (RFRP)], with RFRP-1 and−3 both being RFamide peptides, while RFRP-2 is not (20). Since the initial discovery of these RFamide-related peptides in mammals, most findings in reproductive biology have focused on RFRP-3 as the mammalian ortholog of GnIH. As described further below, studies across mammalian species indicate a pronounced role for this neuropeptide in regulating reproductive function.

The receptor for GnIH/RFRP-3 is a G-protein coupled receptor (GPR), originally named OT7T022 (21), but now more commonly referred to by name of the receptor for which it was found to be identical, the formerly-orphaned GPR147. Around the same time as this discovery, two receptors for another RFamide-peptide, neuropeptide FF, were identified and called NPFFR1 and NPFFR2 (22). NPFFR1 was found to be identical to GPR147, whereas NPFFR2 was identical to another GPR, GPR74. GPR147 has a high affinity for GnIH/RFRP-3 whereas NPFF exhibits potent agonistic activity at GPR74 (16, 22–24). Together, these findings revealed GPR147/NPFFR1 as the GnIH/RFRP-3 receptor. GPR147 most-commonly couples to an inhibitory G protein (Gαi), with GnIH/RFRP-3 suppressing cAMP activity (21, 25). However, in some instances, GPR147 is coupled to Gαs or Gαq proteins (26), where this differential coupling may account for disparity in the effects of RFRP-3.

As indicated previously, in most rodents, RFRP-3 perikarya are restricted to the DMH (8, 9, 27), although, in rats, a significant number of cells are observed in the region between the DMH and ventromedial nucleus of the hypothalamus (VMH) (21, 28). In mammals, RFRP-3-immunoreactive (-ir) fiber projections are extensively scattered throughout the diencephalon, mesencephalon and limbic structures (29–32), providing divergent neural pathways to broadly influence neurophysiology and behavior.

### Evidence for a Role of RFRP-3 in Reproduction

As suggested previously, RFRP-3 generally inhibits gonadotrophin synthesis and/or secretion across mammals, including humans (27, 30, 33–35). RFRP-3 acts directly and indirectly to influence GnRH cell function. For example, RFRP-3 cell fibers form close contacts with GnRH cells and around a third of GnRH cells express GPR147, pointing to direct actions of RFRP-3 on the GnRH system (17, 36–38). Likewise, RFRP-3 inhibits cellular activity in about 40% of GnRH cells *in vitro* (39, 40). RFRP-3 may also act to suppress GnRH cellular activity via kisspeptin cells, as RFRP-3 cell projections form close connections with kisspeptin neurons in mice, sheep and monkeys (37, 41, 42), with a small percentage of kisspeptin cells in the anteroventral periventricular nucleus (AVPV), and ~25% of kisspeptin cells in the arcuate nucleus, expressing GPR147 in mice (36, 42).

In some cases, however, RFRP-3 stimulates gonadotropin secretion, with differences observed based on sex, season or reproductive status. For example, in male Syrian hamsters (*Mesocricetus auratus*), RFRP-3 increases GnRH neuronal activity (i.e., increases *c-Fos* expression) and increases gonadotropin and testosterone release (43). This pattern differs from that observed in female Syrian hamsters where RFRP-3 suppresses LH if administered around the time of the LH surge (30, 44). Similarly, in male mice, RFRP-3 stimulates LH secretion, at least in part, via actions on kisspeptin as the stimulatory effect of RFRP-3 is diminished in kisspeptin receptor knockout mice (45). In female mice, as in Syrian hamsters, RFRP-3 inhibits LH when estradiol concentrations are high around the time of the LH surge, but is without effect during diestrus or in ovariectomized females with low estradiol concentrations provided exogenously (45). Finally, in male Siberian hamsters (*Phodopus sungorus*), RFRP-3 stimulates LH secretion in short-day, reproductively-inhibited hamsters, but inhibits LH secretion in long-day, reproductively-competent animals (17). Together, these findings confirm a role of RFRP-3 in the central control of reproduction, but its effects are dependent on species, sex, reproductive status and hormone concentrations, which all likely affect the specific G-protein to which GPR147 is coupled. Surprisingly, however, GPR147/NPFFR1 female null mice exhibit moderate reproductive phenotypes with larger litter, and increased arcuate kisspeptin synthesis, higher serum FSH concentrations, and augmented LH responses to GnRH (46). The disparate results in the effects of GPR147/NPFFR1 inactivation and exogenous administration of RFRP-3 may be explained by compensatory mechanisms by other RF-amide systems.

### Potential Roles of RFRP-3 on the Pituitary and Gonads

In addition to actions on GnRH neurons, RFRP-3 may alter gonadotropin synthesis and secretion via the pituitary, although findings are disparate across studies and species. For example, RFRP-3 projections have been shown to project to the outer layer of the median eminence [hamsters (47), sheep (33), macaque (37), and humans (38)]. In contrast, using peripheral injections of fluorogold to label hypophysiotropic cells, RFRP-3 cells were not labeled in rats (48). In other studies, RFRP-3 terminal fibers in the median eminence are sparse or absent [mice (49); brushtail possum (50); macaque (51)]. Although results are equivocal regarding projections to the median eminence across species, GPR147 is expressed in the pituitary of hamsters (47) and humans (38) and RFRP-3 inhibits gonadotropins in cultured pituitaries from sheep (52), cattle (53), and rat (54). In ewes, RFRP-3 is detected in hypophyseal portal blood and exogenous RFRP-3 has been reported to significantly reduce the GnRH-induced LH response (55). In another study, however, peripheral administration of RFRP-3 in ewes was unable to inhibit pulsatile LH secretion or the E2-induced LH surge (56), raising the question of whether or not RFRP-3 acts on pituitary gonadotropes despite being detectable in portal blood.

In addition to potential actions at the level of the pituitary, RFRP-3 is produced, and appears to act locally, to regulate gonadal function. Early work discovered that GnIH is synthesized in ovarian granulosa cells and in the testicular interstitial layer and seminiferous tubules of birds (57). Moreover, in birds, GnIH application decreases testosterone release from gonadotropin-stimulated testes *in vitro*, pointing to a functional role for gonadal GnIH (58). Later, it was shown that RFRP-3 is synthesized in the gonads of all mammals studied to date (59), including humans (60), non-human primates (61), Syrian hamsters (62), mice (60, 63), rats (64), ewe (65), and pigs (66). Across species, the gonads synthesize RFRP-3 and GPR147 (57, 59–61, 63). In mice, testicular RFRP-3 synthesis increases during reproductive senescence possibly contributing to aging-related decrements in testicular functioning (67). In human granulosa cell cultures, RFRP-3 inhibits gonadotropin-induced intracellular cAMP accumulation and progesterone secretion (60). Finally, RFRP-3 and GPR147 are synthesized in ovarian granulosa cells and antral follicles during proestrus and estrus and in luteal cells during diestrus in mice (63), suggesting participation in follicular development and atresia. Together, these findings suggest that GnIH/RFRP-3 is commonly synthesized in the gonads across species and may act locally to fine-tune gonadotropin-regulated gonadal functioning.

## RFRP-3 Contributes to the Daily Rhythm of Reproduction in Female Rodents

### Daily and Ovarian Rhythms in Female Reproduction

Successful female reproduction requires the activation of specific neuronal and hormonal pathways in order to synchronize ovulation with maximal locomotor activity and optimal arousal state. Female mammals display rhythms of different, recurrent time scales that range from minutes (pulsatile GnRH release) to hours/days (LH surge), days/weeks (ovarian cycle) or even months (seasonal reproduction).

Ovarian activity displays regular cycles (~28 days in women and 4–5 days in rodents) driven by changes in circulating levels of the pituitary gonadotropins LH and FSH. During the first stage of the ovulatory cycle (follicular phase in humans, metestrus-diestrus in rodents), FSH secretion gradually increases, promoting ovarian follicular development. In turn, maturing follicles secrete increasing concentrations of E2. The second stage of the reproductive cycle (luteal phase in women; proestrus-estrous in rodents) is immediately preceded by a pronounced and transient rise in LH secretion (surge) that initiates the release of mature oocyte(s) from ovarian follicles. The generation of the LH surge requires high circulating levels of E2, indicative of follicle maturation, as well as a daily signal, ensuring that ovulation occurs at the right arousal time to optimize breeding success. Indeed, the LH surge occurs at a specific time of day, corresponding to the end of the inactive phase, thus in late afternoon in nocturnal rodents (e.g., mice, rats, hamsters) and early morning in diurnal species (e.g., Nile grass rat, humans) (68).

Most biological functions exhibit daily rhythms that are coordinated by a complex network of endogenous central and peripheral circadian clocks synchronized to the 24 h light-dark cycle (69). In mammals, the suprachiasmatic nucleus (SCN) is the main pacemaker that orchestrates the circadian timing system as it drives central and peripheral oscillators and ultimately coordinates daily rhythms in physiology and behavior. Exploring the pathways by which the circadian clock synchronizes GnRH neuronal activity and upstream modulatory systems is essential to fully understand the mechanisms of female reproduction. Indeed, circadian disruption has been associated with various abnormalities in fertility and reproduction. Early studies in the 50's demonstrated that chemical blocking of neural clock output alters the LH surge in female rats (70, 71) and hamsters (72). Furthermore, SCN lesions cause anovulation in female rats, presumably resulting from the loss of diurnal variation in the sensitivity of the reproductive axis to E2 positive feedback (73). Finally, female mice deficient for the clock gene, *Clock*, exhibit abnormal estrous cycles, do not have a detectable LH surge on the day of proestrus, and generally fail to carry pregnancies to term (74). Similarly, women with single-nucleotide polymorphisms in the circadian clock gene *ARNTL* exhibit more miscarriages than those without such mutation (75).

It appears that the circadian signal is sent to the reproductive system each day, but its impact is masked by low circulating E2. Thus, in female rodents provided with chronic, proestrus-like concentrations of E2, daily LH surges are observed for several consecutive days, revealing the circadian mechanism underlying surge generation (76–78). Altogether, these findings, largely obtained in female rodents, indicate that the timing of the preovulatory LH surge is strictly time-gated by a combination of daily and ovarian signals. Although the daily signal is communicated each day by the SCN to the GnRH/LH pathway, E2 secretion from mature oocytes needs to reach a certain threshold in order to exert positive feedback on the hypothalamo-pituitary-gonadal axis and allow the generation of the LH surge.

### Mechanisms Regulating the Circadian-Estrogen Sensitive Preovulatory LH Surge

Two principal SCN neurotransmitters, vasoactive intestinal peptide (VIP), and arginine-vasopressin (AVP), are thought to be implicated in relaying daily cues to GnRH neurons and therefore controlling the timing of the preovulatory LH surge.

VIP content in the rat SCN displays daily variation which is abolished under constant darkness, suggesting that VIP is implicated in the transmission of photic information (79). Furthermore, the daily rhythm in SCN VIP appears sex-dependent since *VIP* mRNA levels peak during the light phase in female rats but during the dark phase in male rats (80). The observation that a central blockade of VIP signaling decreases the LH surge in female rats indicates a role of this peptide in female reproduction (81, 82). Indeed, ~45% of the GnRH cells are innervated by VIP-containing fiber terminals and unilateral thermal lesions of the majority of SCN VIP cells results in a 50% decrease of VIP nerve contacts on GnRH cell bodies on the lesioned side, compared to the intact side, of the brain (83). Furthermore, the use of anterograde tracing demonstrated a direct connection between the SCN and GnRH neurons (84). Interestingly, there is a sex-dependent difference in the VIP-GnRH pathway, with the number of VIP terminals onto GnRH neurons, and the percentage of GnRH neurons contacted by VIP fibers, being higher in females compared to males (85). About 40% of GnRH neurons express VIP2 receptor (86) and exogenous VIP application to brain slices increases GnRH neuron action potential firing and intracellular calcium (87, 88), supporting the idea that VIP may provide a direct excitatory signal from the SCN to the GnRH system.

*AVP* expression exhibits both circadian and daily variation in the SCN (89). AVP release in the SCN vicinity has been found to peak during midday while minimum release occurs at midnight (90). Unlike VIP, no sex-dependent differences in *AVP* gene expression are found in the SCN (80). Increasing evidence indicates that the rhythm in SCN AVP release is critical for the daily timing of the preovulatory LH surge. Indeed, central administration of AVP in OVX, E2-treated rats, bearing complete SCN lesions, is sufficient to trigger a LH surge (91). However, the ability of AVP to trigger the surge is time-dependent, with administration during the latter half of the light period, but not the first half, being effective (92). Moreover, central administration of a V1a receptor antagonist decreases LH surge amplitude in rats (93). Finally, in *Clock* mutant female mice, central injections of AVP can restore a preovulatory-like LH surge (74). Unlike VIP, SCN AVP neurons appear to regulate the GnRH/LH surge indirectly via kisspeptin neurons located in the preoptic area (AVPV in rodents), a highly sex-dimorphic brain area (94, 95). Thus, in female rodents, AVPV kisspeptin neurons receive direct SCN-derived AVP inputs and express V1a receptors (96, 97), and direct application of AVP to brain slices increases neuronal firing and intracellular calcium concentrations in AVPV kisspeptin cells (98). Importantly, AVPV kisspeptin neurons display ERα, and E2 not only potently stimulates kisspeptin synthesis (13, 94, 95), but is also required for the AVP-induced activation of kisspeptin cells (98). Finally, activation of AVPV kisspeptin neurons coincides with the time of LH surge, during the sleep/wake transition in proestrus or in OVX E2-treated female rodents, but does not display daily rhythms during diestrus or in OVX animals (97, 99, 100).

Therefore, data primarily obtained in female rodents indicate that both SCN-derived VIP fibers acting directly on GnRH neurons, and AVP fibers acting indirectly via preoptic kisspeptin neurons, are involved in the timing of the preovulatory LH surge. In addition to this mechanism of surge control, as described further below, RFRP-3 neurons may also be part of the pathway relaying daily time cues from the SCN to GnRH neurons in order to time the preovulatory LH surge.

### Evidence for a Role of RFRP-3 Neurons in the Daily Timing of the LH Surge

Earlier work in female Syrian hamster reported a monosynaptic connection between the SCN and RFRP-3 neurons, suggesting that RFRP-3 neurons are regulated by the SCN clock (47). In accordance with this hypothesis, a daily rhythm in RFRP-3 neuronal activity has been reported, with a lower number of RFRP-3 neurons expressing c-FOS coincident with the timing of the LH surge in female Syrian hamsters (44, 47) and mice (101). Equivocal findings are reported regarding the association between RFRP-3 cell activation state and the number of *Rfrp* expressing neurons, with daily variation in RFRP-3 neuronal activity being associated (47) or not (44, 101), with corresponding changes in the number of *Rfrp* expressing cells. As in Syrian hamsters, *Rfrp* expression is decreased during the preovulatory period in ewes (102). The role of RFRP-3 neurons in relaying circadian information to GnRH neurons is further supported by an experimental protocol where female hamsters kept under constant light conditions split their locomotor activity and exhibit two daily LH surges. In these conditions, the two halves of SCN oscillate in antiphase and RFRP-3 neurons are active asymmetrically in opposition to GnRH neuron activation (47).

A recent study in female Syrian hamster demonstrated that AVP- and VIP-ergic fibers from the SCN form close appositions with RFRP-3 neurons and that central injection of VIP decreases RFRP-3 neuronal activity in a time-dependent manner, being effective in the afternoon, but not in the morning, while central AVP has no significant effect (103). It is yet unclear, however, whether the action of VIP on RFRP-3 neurons is direct or not since < 10% of RFRP-3 neurons appear to express the *VPAC1* and *VPAC2* receptors (103). Altogether, these findings suggest a SCN-derived VIP daily regulation of RFRP-3 neuronal activity, at least in Syrian hamsters. Additionally, there is evidence in female rodents that RFRP-3 neurons, similar to kisspeptin neurons (100), are able to keep track of time intrinsically, expressing the clock protein PER1 with a peak at ZT12 (103).

Unlike kisspeptin cells, it is likely that high circulating levels of E2 are not required for the daily rhythm in RFRP-3 neurons as daily rhythms in RFRP-3/c-FOS are similar during diestrus and proestrus in one study in Syrian hamsters (44). Although another report indicates that daily variation is abolished in OVX hamsters and restored in OXV+E2 animals (47), this study investigated different time points and used a different protocol that might account for the disparity between findings.

**Figure 1:**
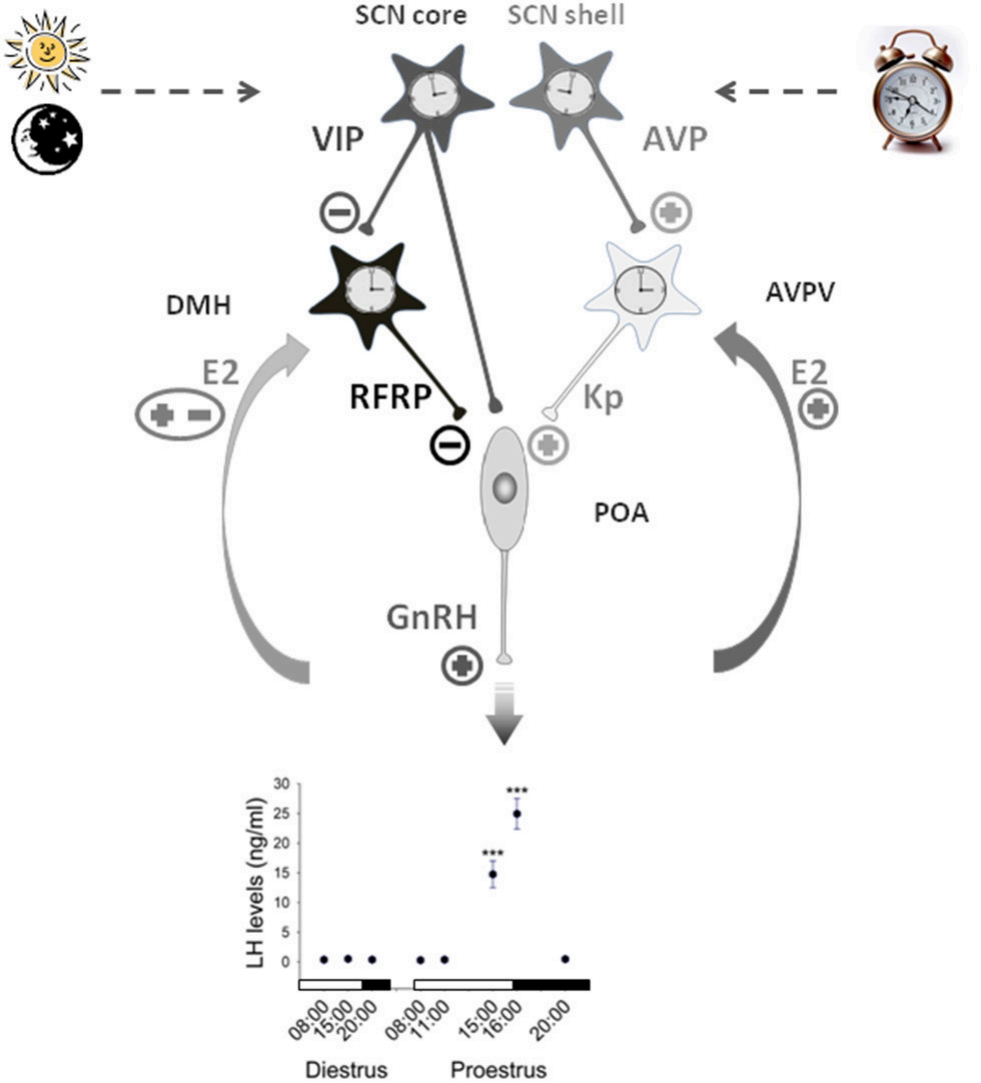
Working model illustrating the contribution of RFRP-3 neurons in the central control of the daily gating of the preovulatory LH surge in female rodents. Neurons of the suprachiasmatic nuclei (SCN) synthesizing vasopressin (AVP) and vasoactive interstinal peptide (VIP) exhibit daily variation controlled by an intrinsic circadian clock and the daily change in light input. The SCN VIP output time GnRH neurons activity either directly or via the RFRP3 neurons located in the dorsomedial hypothalamus (DMH) which further inhibit GnRH neurons at the light/dark transition. The SCN AVP output activates GnRH neurons through the stimulation of neurons located in the anteroventral periventricular nuclei (AVPV) and releasing the potent stimulatory peptide kisspeptin. Additionally kisspeptin neurons receive a positive estradiol (E2) feedback on the day of proestrus while the effect of E2 on RFRP3 neurons is still unclear. This coordinated pathway is proposed to trigger a preovulatory GnRH/LH surge at the light/dark transition of the proestrus stage [LH data adapted from (105)].

A number of studies are consistent with an inhibitory action of RFRP-3 on LH secretion in female mammals (30, 104). In Syrian hamsters (44) and mice (45), central RFRP administration decreases LH secretion when given around the time of the preovulatory LH surge, whereas it has no effect when given at other time points where LH secretion is low (early day of proestrus or diestrus). Therefore, the inhibitory effect of RFRP-3 on LH secretion, associated with decreased activity of RFRP-3 neurons in late afternoon, possibly mediated by a SCN VIPergic signal, indicates that tonic RFRP-3 inhibitory input is lifted at the time of the preovulatory LH surge (Figure 1).

### The Controversy of E2 Feedback on RFRP-3 Neurons

The possibility that RFRP-3 neurons, similar to kisspeptin neurons (68), may be a central site for the E2 feedback has been widely studied. However, the results obtained in different species, sex and conditions are conflicting.

ERα are found in 40 and 25% of RFRP-3 neurons in female Syrian hamsters (30) and mice (19, 106), respectively. Studies have reported that E2 treatment in OVX Syrian hamsters increases c-FOS expression in RFRP-3 neurons (30) while others, in contrast, show that E2 treatment decreases the amount of *Rfrp* mRNA per cell and the total amount of *Rfrp* mRNA in both male and female mice (106). In female rats, RFRP-3 neuronal activity is reported to be higher during diestrus compared to proestrus and estrous (107), suggesting a role for E2 in RFRP-3 cell activational state across the ovulatory cycle in this species. Finally in female rats (108) in male (109) and female (44) Syrian hamsters, and in male Djungarian hamsters (110), gonadectomy with/without sex steroid replacement does not have a significant effect on RFRP-3 synthesis.

Other experimental paradigms indirectly suggest a possible influence of E2 on RFRP-3 neurons. For example, E2 treatment increases RFRP-3 synthesis in the hypothalamic mHypoA-55 rat cell line (111). In Syrian hamsters, food-restriction increases the percentage of RFRP-3 cells expressing *c-Fos*, with increased ovarian steroids at the time of estrus abolishing the impact of food restriction on RFRP-3 cellular activation (112). Finally, in female rats, RFRP-3 synthesis varies according to reproductive stage, with increased levels at the time of puberty when the endogenous sex steroid levels are highest (108).

### Concluding Remarks on the Role of RFRP-3 in the Daily Timing of the LH Surge in Female

Female reproduction is cyclic and in female mammals, possibly including women although this is still controversial, daily time cues are integrated within the reproductive system to coordinate the LH surge and consequential ovulation with the best period of the day. The hypothalamic SCN clock plays a key role in conveying daily information to the reproductive system, and increasing evidence indicates that RFRP-3 neurons, in addition to kisspeptin neurons, are a key relay between the SCN clock and GnRH neurons. Recent data indicate that the SCN-derived VIP output drives RFRP-3 neuronal activity, but the mechanisms involved are still unclear. Furthermore, while numerous studies now agree on the critical role of kisspeptin in the timing of LH surge, the specific significance of RFRP-3 on the occurrence of the LH surge requires further investigation.

## RFRP-3 Plays a Role in Seasonal Reproduction

### Seasonal Rhythms in Reproduction, the Role of Melatonin and Thyroid Hormones

The marked changes in environmental factors throughout seasonal cycles require species to display predictive adaptation of their behavior and physiology to survive. Notably, many mammalian species synchronize their reproductive activity with one particular time of the year so that depending on the duration of female gestation, offspring are born at the most favorable period of the year, usually in spring when temperature, humidity and food availability are optimal (113). Thus, two categories of breeders are described depending on the mating period: long-day (LD) breeders like rodents with a few weeks of gestation and short-day (SD) breeders like sheep, goats, or deer, with a few month of gestation (114).

Since the 60's, it has been known that the pineal hormone melatonin is a major signal for the synchronization of reproduction with the seasons. Indeed, melatonin synthesis and release occurs only during the night and, therefore, the nocturnal production of melatonin is longer in the autumn/winter SD as compared to spring/summer LD (115). Hoffman and Reiter were the first to demonstrate that the elimination of this neuroendocrine calendar by pinealectomy abolishes the reproductive response of Syrian hamsters to the photoperiod signal (116). It was later established through timed melatonin-infusion experiments that the duration of circulating melatonin, and not its concentration or phase, is the crucial variable triggering photoperiodic adaptations in all seasonal species (114, 117). Intriguingly, although the mechanism is unknown, the same photoperiodic melatonin signal has an opposite reproductive effect on LD and SD breeders. Further, the exact neuroendocrine mechanisms through which the melatonin signal reaches the hypothalamic-pituitary-gonadal (HPG) axis are yet not fully understood.

Neuroanatomical approaches identifying melatonin binding sites and studies performing melatonin infusions in lesioned animals have identified hypothalamic areas as putative targets and/or relays of melatonin signaling for the control of seasonal reproduction. According to species, the mediobasal hypothalamus (MBH), the SCN or the premammilary region of the hypothalamus were proposed to be involved in this process (118–121). Until now, however, the means by which melatonin drives seasonal reproduction through actions at these hypothalamic sites remained unknown. Importantly, in numerous mammalian species, melatonin receptor mapping revealed a high density of seasonally regulated receptors in the *pars tuberalis* (PT), the rostral part of the adenohypophysis extending below the median eminence (122–125). These neuroanatomical observations indicated that melatonin does not act directly on the hypothalamus but through a multistep pathway involving the PT (126, 127).

Earlier studies also pointed to the role of the thyroid hormones T3 (triiodothyronine) and T4 (thyroxine) in seasonal reproduction. Notably, thyroidectomy in ewes during the breeding season prevents seasonal LH decline and suppresses reproductive functions (128). Furthermore, two enzymes involved in the metabolism of thyroid hormones, the type 2 deiodinase (Dio2, responsible for converting inactive T4 into bioactive T3) and type 3 (Dio3, responsible for the inactivation of T4 and T3) are synthesized in tanycytes, ependymal cells lining the basal part of third ventricle, with higher *Dio2* and lower *Dio3* expression in LD than in SD, leading to increased levels of T3 in the hypothalamus of LD animals (129–132).

The link between the seasonal changes in melatonin and thyroid hormone was revealed by neuroanatomical, physiological and genome-wide analyses. These studies revealed that most melatonin receptor-synthesizing PT cells also express thyroid stimulating hormone (TSH) in a melatonin-dependent manner with marked inhibition by the long SD peak of melatonin (133–135). Additionally, in quail transferred from SD to LD condition, PT *TSH*β was identified as one of the first genes to be increased, closely followed by a combined increase in *Dio2* and decrease in *Dio3* expression in tanycytes (136). This study also demonstrated that TSH receptors are located on tanycytes and that their activation by TSH increases *Dio2* expression (136). In a contemporary study, a similar direct stimulatory effect of TSH on tanycytic *Dio2* expression was also reported in the sheep (137). In line with these findings, it was demonstrated in TSH receptor knock-out mice that melatonin treatment no longer inhibits *Dio2* and increases *Dio3* expression in tanycytes (138). Further studies have demonstrated that the photoperiod/melatonin-dependent inhibition of PT TSH associated with a tanyctytic switch in Dio2/Dio3 is highly conserved in seasonal vertebrates (126, 139). Finally, the observation that intra-hypothalamic administration of TSH or T3 can restore a LD seasonal phenotype in various seasonal breeders (130, 140–143) led to the functional model where melatonin communicates the seasonal message through PT TSH-driven regulation of hypothalamic T3 which, in turn, regulates seasonal physiology (127).

One of the current challenges is to identify the molecular and cellular targets through which hypothalamic T3 synchronizes biological functions, notably reproduction, with the seasons. This issue requires analyzing if and how the central structures known to regulate these functions are regulated by the melatonin/T3 signal.

### Evidence for a Role of RFRP-3 in the Seasonal Rhythm of Reproduction

#### RFRP-3 Neurons Are Regulated by Photoperiod Through the Melatonin Signal

In early studies, it was shown that GnIH (15) and RFRP-3 (30) in seasonal quail and rodents, respectively, are synthesized in hypothalamic neurons and are able to alter LH release, altogether indicating that this peptide may be involved in the seasonal regulation of reproduction.

The first studies on quail and sparrow reported seasonal variation in GnIH synthesis which correlated with seasonal changes in reproduction (144, 145). Additionally, melatonin administration, in pinealectomized and enucleated (pineal gland and eyes removed to eliminate all sources of melatonin) quail, was shown to act directly on GnIH neurons to inhibit GnIH synthesis in a dose-dependent manner (18).

Subsequently, it was found that, in seasonal rodents, the number of RFRP-3 neurons in the dorso/ventromedial part of the MBH displays marked photoperiodic changes (109). Indeed RFRP-3 synthesis is higher in LD-adapted, sexually active animals as compared to SD-adapted sexually inactive male Syrian and Siberian hamsters (109, 146). Like in birds, although in an opposite manner, seasonal variation in RFRP-3 synthesis depends on melatonin since pinealectomy increases, and injection of melatonin decreases, the number of RFRP-3 expressing neurons in hamsters (17, 109). Additionally, expression of *GPR147* in various hypothalamic areas (31) and the number of GnRH cell bodies receiving RFRP-3 fiber contacts (17, 32) are increased in LD hamsters. Notably, manipulating testosterone levels by castration with/without testosterone supplementation has no significant effect on the photoperiodic regulation of RFRP-3 synthesis (109, 110, 146). Likewise, ovariectomy has no effect on RFRP-3 synthesis in ewes (32), and Syrian hamsters (44) thus demonstrating that these changes are driven by melatonin independent of sex steroid feedback.

#### Seasonal Variation in RFRP-3 Expression Is Highly Conserved Among Seasonal Species

**Figure 2:**
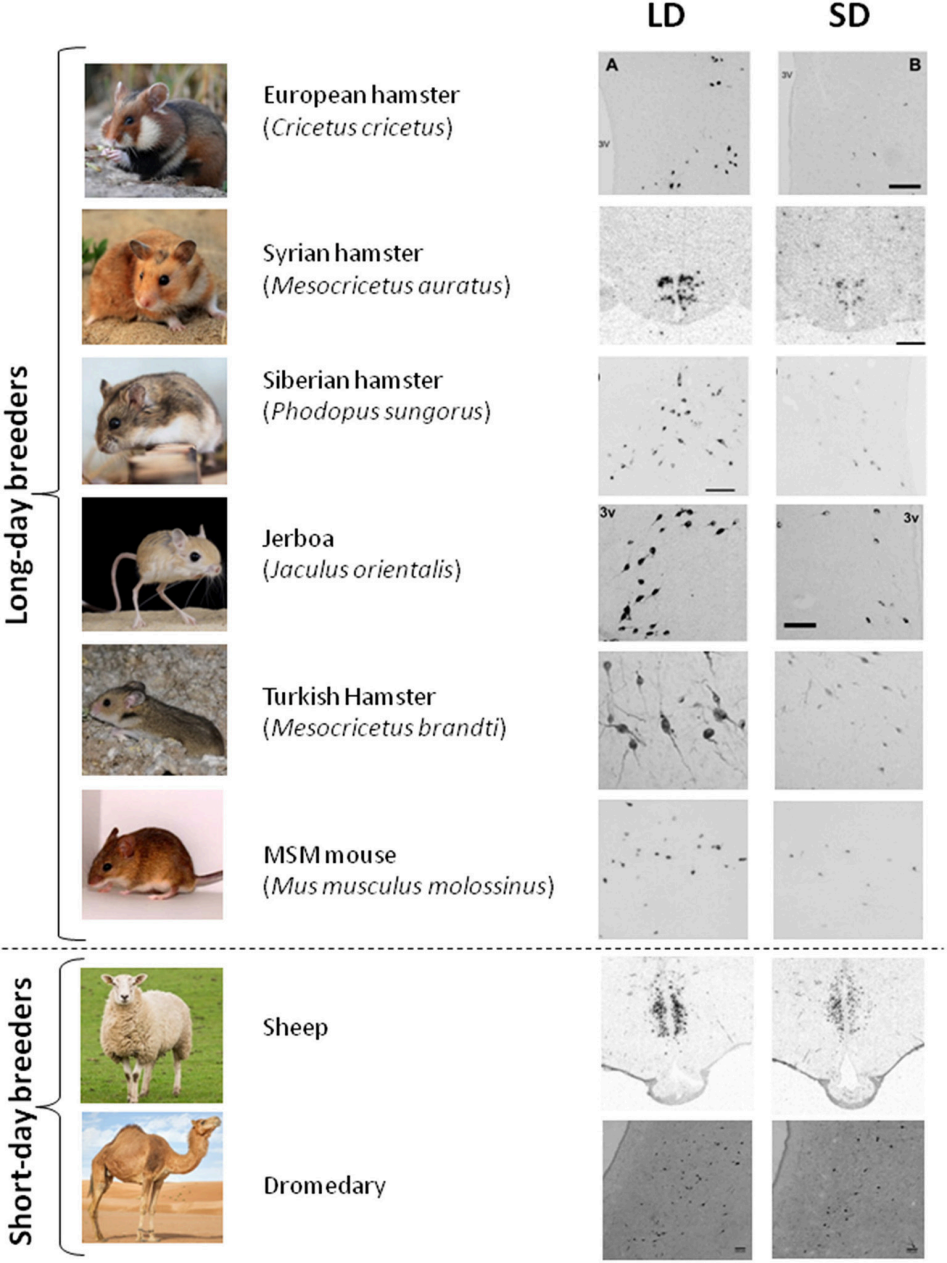
RFRP-3 synthesis in the mediobasal hypothalamus exhibits a conserved seasonal pattern. RFRP-3 expression, attested by the number of neurons or the level of *Rfrp* mRNA, is higher in long-day (LD) condition as compared to the short-day condition (SD), in LD breeders (European hamster, Syrian hamster, Siberian hamster, Jerboa, Turkish hasmter, and MSM mouse) as well as in SD breeders (sheep, dromadary). Adapted from (109) (Syrian hamster), (148) (European hamster), (149) (Jerboa), (147) (Turkish hamster), (150) (sheep), with appropriate permissions obtained from the copyright holders; mouse and dromedary data are from unpublished results.

The seasonal pattern of RFRP-3 synthesis seems well conserved in mammalian LD breeders, with higher RFRP-3 mRNA/protein expression in LD than in SD observed in Syrian and Siberian hamsters (109, 146), Turkish hamsters (147); European hamsters (148) and Jerboa (149). Notably, melatonin proficient mice such as *Mus muculus mollosinus* (unpublished results) and CBA (unpublished results, Figure 2) exhibit photoperiodic variation in RFRP-3 synthesis with higher values in LD conditions. Interestingly, in SD breeders like sheep (32, 151), the brushtail possum (50), goats (152), and camels (unpublished results), RFRP-3 synthesis is also elevated in LD even though these animals are sexually inhibited. Finally, in striped hamsters (*Cricetulus barabensis*), the highest expression of *Rfrp* is observed in breeding males, whereas breeding females exhibit lowest mRNA expression, pointing to disparate seasonal roles of RFRP-3 between the sexes (153).

Altogether these data demonstrate that seasonal/photoperiodic variation in RFRP-3 expression is highly conserved among seasonal mammals with higher levels in LD summer as compared to SD winter, whether animals are LD or SD breeders (Figure 2). These findings indicate that melatonin uses similar mechanisms to regulate RFRP-3 expression in all seasonal species and that the switch driving LD or SD breeding activity may be downstream of RFRP-3 neurons.

#### Species-Dependent Effect of RFRP-3 on Seasonal Reproduction

Early studies in quail (15, 145) followed by those in female Syrian hamsters (30) and ewe (33) all indicated an inhibitory effect of GnIH/RFRP-3 on LH secretion. However, analogous to the varying impact of RFRP-3 based on stage of the ovulatory cycle, further studies reported that the role of RFRP-3 in seasonal mammals is more complicated than expected.

Indeed, in male LD-adapted Syrian hamsters, acute injection of RFRP-3 was found to increase LH, FSH and testosterone secretion (43). Furthermore, chronic central infusion of RFRP-3 in SD-adapted, sexually inhibited male Syrian hamsters restores gonadal activity to that of hamsters kept in LD conditions (43). Intriguingly, despite an acute inhibitory effect of RFRP-3 on the preovulatory LH surge in LD-adapted female Syrian hamsters, a chronic central infusion in sexually inactive SD-adapted females fully restores reproductive activity, as observed for male hamsters (44). Even more complexity was revealed following studies of closely-related male Siberian hamsters, where the effect of RFRP-3 on LH secretion depended on photoperiod, with RFRP-3 being stimulatory in SD-adapted and inhibitory in LD-adapted animals (17). In ewes, the first studies reported that RFRP-3 inhibits gonadotropin secretion (33, 52). However, a more recent study using different protocols of RFRP-3 administration could not find any effect on LH secretion in ewes (56).

Therefore, although the melatonin-dependent photoperiodic regulation of RFRP-3 neurons is well conserved among seasonal species, the role of RFRP-3 on the seasonal regulation of reproduction is unclear and appears to be species dependent. Data so far, however, are insufficient to conclude that RFRP-3 is responsible for the LD or SD breeding activity in seasonal species. At this point there is no explanation as to why RFRP-3 displays differential reproductive effects. One hypothesis is that RFRP-3 may bind to different receptors, notably those of the large family of RF-amide peptides known to have cross-binding capacity (154) or interact with different G proteins. Another hypothesis is that RFRP-3 uses intermediate neuronal populations with different downstream effects on GnRH neuron activity and gonadotropin secretion. Notably, RFRP-3 neurons have been reported to project to kisspeptin neurons (36) and the stimulatory effect of chronic RFRP-3 infusion in SD-adapted male (43) and female (44) Syrian hamsters is associated with a marked increase in *Kiss1* expression. Further studies are needed to better understand the downstream effect of RFRP-3 on GnRH neurons and gonadotropin secretion and reveal why this neuropeptide displays opposite actions according to species, sex, and photoperiod.

### Are RFRP Neurons the Site of Seasonal Changes in Hypothalamic TH?

**Figure 3:**
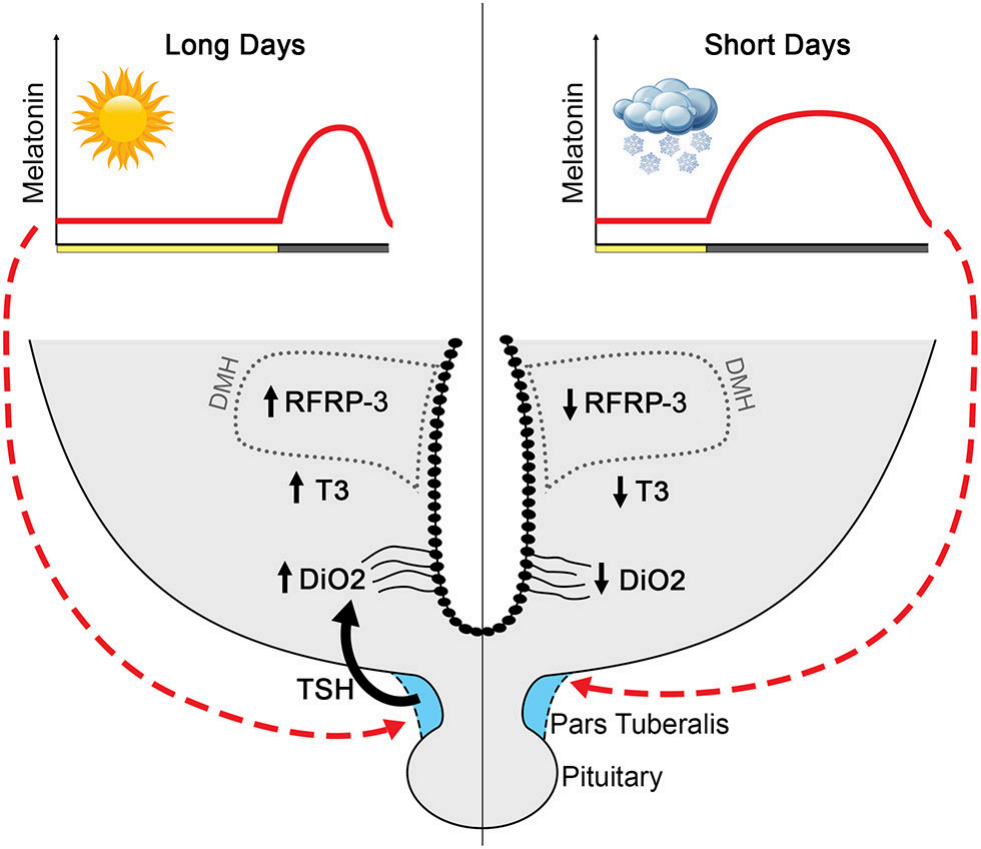
Proposed model by which melatonin influences RFRP-3 and the reproductive axis in long-day-breeding rodents. Melatonin, coding for day length, acts on melatonin receptors in the pars tuberalis to influence TSH production (133–135). The long duration melatonin signal in short days suppresses TSH production, whereas short duration melatonin during long days is associated with robust TSH release. In long days, TSH stimulates tanycytes to upregulate *Dio2* and, consequently, increases T3 (129–132, 142). Long days are associated with high RFRP-3 expression, with neuron numbers markedly reduced in short-day animals. Although T3 increases expression of RFRP-3 in short-day animals to resemble that of long-day rodents (142), whether RFRP-3 cells are direct targets of T3 remains to be determined.

The conserved photoperiodic regulation of RFRP-3 leads to the hypothesis that melatonin may use the PT TSH/hypothalamus T3 pathway to regulate RFRP-3 synthesis (Figure 3). Indeed, chronic central infusion of TSH in SD-adapted Syrian and Siberian hamsters restores RFRP-3 expression similar to that observed in LD animals, and this effect is associated with a restoration of the LD-phenotype of kisspeptin expression and gonadal activity (142). Furthermore, another study similarly reported that exogenous T3 injection also results in a LD-like restoration of the number of RFRP-3 and kisspeptin cells as well as testis size (155). Altogether these data suggest that seasonal changes in hypothalamic T3 could act directly on RFRP-3 neurons. However, no data to date have demonstrated the localization of T3 receptors in RFRP-3 neurons and a direct effect of T3 on these neurons. Further, the possibility of an indirect effect should not be excluded.

Even if the photoperiodic regulation of RFRP-3, together with its reproductive effect, is consistent with the seasonal pattern of reproductive function, the causal/temporal relation between seasonal changes in RFRP-3 and reproduction is still questionable. Indeed in photorefractory hamsters undergoing spontaneous gonadal recrudescence after long SD exposure, RFRP-3 expression stays low, reflecting photoperiod rather than reproductive state (109). Further, in a recent study following the coordinated dynamic changes in RFRP-3 and reproductive parameters, it appears that the LD-induced activation of gonadotropin production precedes an increase in RFRP-3 (156). Thus, although exogenous RFRP-3 has been reported to restore gonadal activity in SD-adapted hamsters (43, 44), its role in initiating the neuroendocrine cascade leading to reactivation of the reproductive axis at breeding season remains to be fully understood.

### Concluding Remarks on the Role of RFRP-3 in Seasonal Reproduction

So far all studies have demonstrated that RFRP-3 expression in the MBH is inhibited by the SD pattern of melatonin. Because RFRP-3 has the capacity to differentially alter GnRH neuronal activity and LH secretion, this neuronal population appears as a key candidate to regulate the downstream reproductive pattern of a LD- or a SD- type of breeder according to gestational status. However, several aspects remain to be clarified to support this hypothesis. First, in contrast to kisspeptin which exhibits a well conserved stimulatory effect on GnRH neuronal activity, RFRP-3 has photoperiod, sex- and species-specific effects making difficult to understand its exact role and contribution to seasonal reproduction. Secondly, it is yet unclear whether RFRP-3 neurons are the primary targets of melatonin-dependent changes in hypothalamic T3 to relay seasonal cues to the GnRH neurons.

## General Conclusions

The studies reviewed herein demonstrate that RPRP-3 neurons display both daily and seasonal variations in numerous species, thus supporting a role for this hypothalamic peptide in the integration of geophysical cues.

Although RFRP-3 is consistently reported to regulate reproductive axis function, the effect on GnRH neuronal activity and gonadotropin secretion is highly dependent on species, sex and environmental conditions. This complexity in the impact of RFRP-3 has hampered the ability to determine the precise role of this neuropeptide in the synchronization of the preovulatory LH surge in females and long-term breeding in seasonal species. Clearly, highly specific pharmacological tools [such as GJ14 (157) or RF313 (158)], new cellular models and novel genetically modified rodent models are required to better understand the physiological role of RFRP-3 in reproductive rhythms.

Determining the roles of RFRP-3 is further complicated by increasing evidence indicating that RFRP-3 is a pleiotropic peptide involved in functions other than reproduction, notably metabolic activity and stress regulation (8, 159, 160). Because reproduction is modulated by energy state and by stress conditions, it is possible that RFRP-3, at least in part, indirectly regulates reproduction via metabolic- and stress-regulated mechanisms. Food intake and metabolic activity, for example, display major circadian and seasonal changes in mammals which may interfere with reproductive cycles. Indeed, metabolic alterations such as food restriction or obesity are known to impair reproduction. As RFRP-3 increases food intake in various species, possibly through actions on orexigenic NPY neurons (34, 161), and food restriction decreases RFRP-3 synthesis in rats and sheep (65, 162), it is possible that RFRP-3 may also impact reproductive activity indirectly via metabolic pathways (163). Likewise, a number of studies report that acute or chronic stress increases RFRP-3 synthesis via increased levels of glucocorticoids (164–166) and this stress-induced increase in RFRP-3 is associated with an inhibition of LH secretion (164). Finally, *Rfrp* gene silencing completely rescues stress-induced infertility in female rats (167) strengthening the implication that RFRP-3 can influence reproductive function via the stress axis.

In summary, although there is much more to learn, findings to date suggest a role for RFRP-3 in the daily and seasonal regulation of reproduction. Whether RFRP-3 mediates these events through direct actions on the reproductive axis, or indirectly via actions on intermediate systems (e.g., stress or metabolic systems), requires further examination. The advent and application of new experimental tools and animal models to more precisely dissect the roles of this neuropeptide will help to further clarify the specific role of RFRP-3 in the LH surge/ovulation and the neural pathways by which melatonin inevitably influences RFRP-3 cell activity.

## Author Contributions

All authors listed have made a substantial, direct and intellectual contribution to the work, and approved it for publication.

### Conflict of Interest Statement

The authors declare that the research was conducted in the absence of any commercial or financial relationships that could be construed as a potential conflict of interest.
